# Advances in Wearable Sensors for Learning Analytics: Trends, Challenges, and Prospects

**DOI:** 10.3390/s25092714

**Published:** 2025-04-25

**Authors:** Huaqing Hong, Ling Dai, Xiulin Zheng

**Affiliations:** 1 Institute of Language Sciences, Shanghai International Studies University, Shanghai 201620, China; huaqing.hong@ntu.edu.sg; 2 Center for Research and Development in Learning, Nanyang Technological University, Singapore 637335, Singapore; 3 Faculty of Education, East China Normal University, Shanghai 200061, China; 4 National Institute of Education, Nanyang Technological University, Singapore 639798, Singapore; 5 Department of Special Education and Counselling, The Education University of Hong Kong, Hong Kong 999077, China; xzheng@eduhk.hk

**Keywords:** wearable sensors, learning analytics, educational technology, human–computer interaction, adaptive learning systems

## Abstract

Wearable sensor technology is increasingly being integrated into educational settings, offering innovative approaches to enhance teaching and learning experiences. These devices track various physiological and environmental variables, providing valuable insights into student engagement, comprehension, and educational environments. However, the extensive and continuous data streams generated by these sensors create significant challenges for learning analytics. This paper presents a comprehensive review of research on learning analytics incorporating wearable technology, systematically identifying methods and approaches that address wearable sensor data challenges. We begin with a systematic review of wearable sensor technologies’ historical development and the current state of sensor data in learning analytics. We then examine multimodal sensor applications in learning analytics and propose research and application trends aligned with educational development needs. Our analysis identifies three key challenges: ethical considerations, explainable learning analytics, and technological and data management issues. The paper concludes by outlining seven future development directions for wearable sensors in educational contexts.

## 1. Introduction

**Figure 1:**
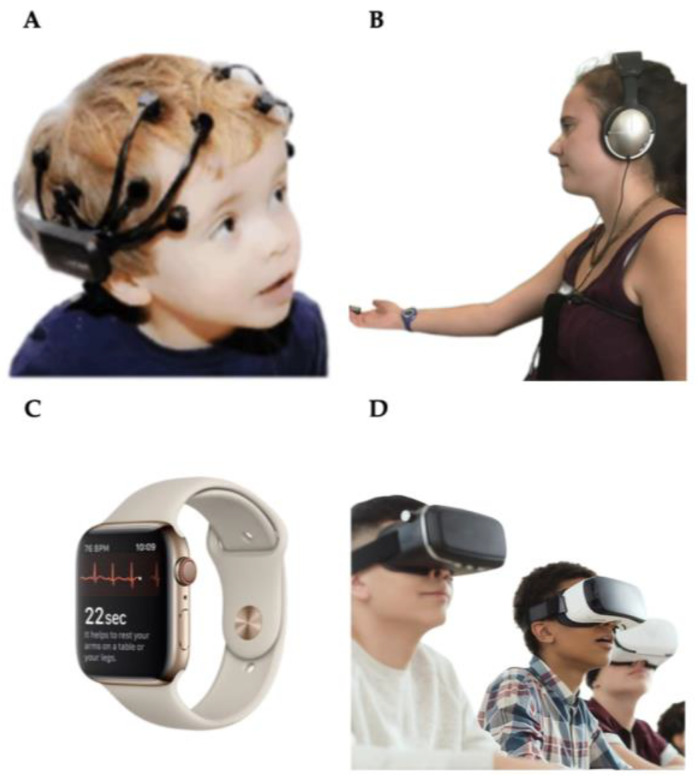
Sensor technologies with smart educational infrastructures and wearable devices create a more adaptive and responsive educational environment: (**A**) head-worn sensors with electroencephalogram (EEG) systems [8]; (**B**) chest-worn sensors [9]; (**C**) wrist-worn sensors [10]; (**D**) head-worn sensors with virtual reality features [3].

In the age of intelligent technologies, the integration of sensor technologies, smart educational infrastructures, and wearable devices marks a transformative shift in learning analytics [1]. These technologies enable the collection and analysis of diverse datasets, including physiological responses, behavioral patterns, and environmental contexts, providing a nuanced understanding of student learning processes [2]. Such insights allow educators and researchers to tailor interventions, refine teaching strategies, and foster personalized learning environments [3]. As illustrated in Figure 1, the interaction between wearable sensors and educational systems facilitates dynamic, data-driven decision-making, advancing both individual and institutional learning outcomes [4]. However, while these advancements hold great potential, they also introduce challenges, particularly regarding data privacy, ethical considerations, and the interpretability of collected data, necessitating critical examination and robust frameworks to address these issues [5]. This paper focuses on biosensors and motion sensors, which have demonstrated significant potential in terms of tracking cognitive engagement, physiological responses, and behavioral patterns in educational settings. By concentrating on these specific sensor types, our review provides in-depth analysis of how they enable adaptive learning analytics, generating data-driven insights that inform personalized educational interventions [6,7].

## 2. Overview of Wearable Sensor Technologies

### 2.1. Wearable Sensor Technologies

Sensor technology facilitates the precise, efficient, and reliable collection of diverse types of information, forming a critical foundation for modern information systems. Fundamental sensor devices include motion sensors, as well as sensors for sound, light, electricity, temperature, humidity, and location, among other applications, as illustrated in Table 1. As a cornerstone of the information society, sensor technology is deeply integrated with sectors such as healthcare, sports, entertainment, industrial production, defense, and traffic management. Current trends in sensor technology emphasize miniaturization, intelligence, and integration. Among the advancements, in-sensor computing has emerged as a transformative approach. This technology integrates data processing capabilities directly into the sensor hardware, enabling localized, real-time analysis without reliance on external systems [1]. Such an approach reduces latency, optimizes energy consumption, and enhances the security and responsiveness of wearable devices. In practical applications, in-sensor computing enables posture and movement analysis in real time, supporting more efficient feedback systems without requiring extensive data transmission. In learning environments, in-sensor computing supports immediate feedback mechanisms by analyzing biometric data, such as heart rate variability and skin conductance, to assess engagement or stress levels during instruction [11]. By processing data locally, these systems overcome the traditional limitations of wearable devices, including delays in feedback and high-power consumption caused by continuous data transmission [12]. Sensors are generally classified into three main categories—motion sensors, biosensors, and environmental sensors—based on their functional applications. Additionally, optical cameras, thermal cameras, and microphones are often included as sensors for motion recognition and affective computing. Wearable motion sensors, small components designed to be placed on various parts of the body, precisely capture and transmit motion data, such as acceleration and angular velocity readings. These sensors are primarily utilized for human activity recognition, with applications spanning healthcare, sports, and smart home environments. Biosensors enable the non-invasive monitoring of physiological data, such as heart rate, respiration, blood pressure, and body temperature. Their ability to provide continuous, real-time tracking makes them essential for health monitoring and medical diagnostics [13]. Environmental sensors capture contextual information about surroundings, including location, light intensity, air quality, noise levels, weather conditions, and atmospheric pressure. These sensors are widely deployed in smart home systems, where they monitor environmental changes to improve quality of life. The integration of these sensor types into wearable technologies offers comprehensive data collection and diverse applications, supporting advancements in fields such as personalized healthcare and adaptive learning analytics.

**Figure 2:**
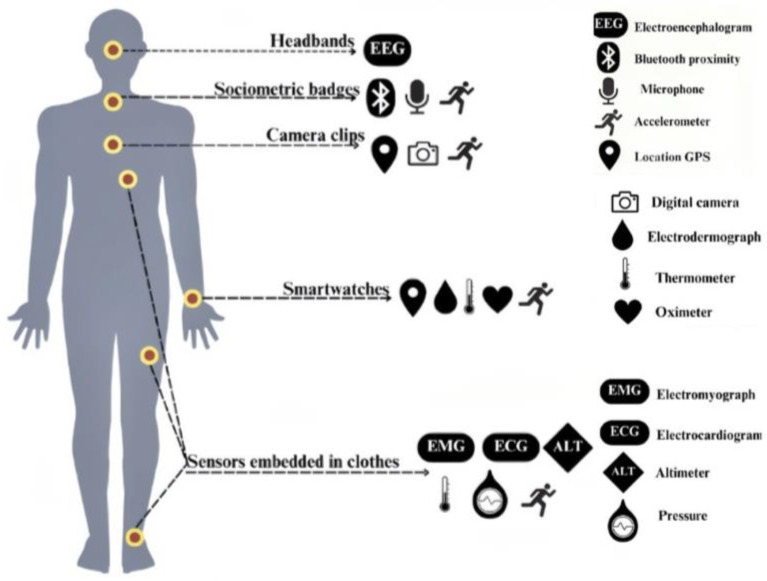
Interaction and coordination with wearable sensor inputs.

As depicted in Figure 2, wearable sensors are embedded with components for activity tracking and monitoring vital signs, functioning as real-time, non-invasive biosensors [22] capable of coordinating multiple sensor inputs [24]. These sensors act as intelligent monitors, offering continuous feedback on the user’s physiological state. Broadly defined, wearable technology includes any body-worn or body-attached device capable of collecting, processing, and providing actionable data. These devices are often described as body-worn information interaction systems [15] and are distinct from traditional self-quantification tools due to their advanced sensory and analytical capabilities. Outfitted with sensitive actuators and sensors, wearable devices collect and store physiological data related to body functions. These data are subjected to cleansing and intelligent processing, enabling the delivery of specific, actionable feedback to users. The defining characteristics of wearable technology are two-fold: the ability to collect and process data, and the integration of embedded systems within wearable devices. Data collection involves the sensing and capture physical activities or physiological characteristics, encompassing processes such as information acquisition, data recording, and advanced analytical processing. This highlights their data acquisition and management capabilities. Embedded wearable systems differentiate themselves from traditional embedded computing through the integration of precise sensors, actuators, and miniaturized storage units within flexible garments or materials. This integration enables seamless human–computer interactions, facilitating real-time data exchange and responsiveness between the user and the device. Wearable technology supports the integration of various sensor types, capable of measuring mechanical (i.e., position, displacement, acceleration, force), acoustic (i.e., volume, tone, frequency), biological (i.e., heart rate, temperature, neural activity, breathing rate), optical (i.e., refraction, light wave frequency, brightness), and environmental (i.e., temperature, humidity) information. This “sensing” capability allows wearable devices to adapt dynamically to their owner, location, and activity, ensuring responsiveness and personalized functionality.

### 2.2. The History of Wearable Sensor Technologies

The origins of wearable technologies can be traced back to the 16th-century innovations of pocket and wristwatches. The evolution of wearable sensors spans three distinct phases: the initial stage, the multifunctional integration phase, and the smart technology phase. The initial stage began with John Harrison’s portable timepiece in 1762 [8], followed by early mechanical wearables like the abacus ring, which combined utility with fashion [11]. A significant milestone came in 1982 with the Seiko Pulsar NL C01, one of the first digital wristwatches with a built-in calculator, which pioneered computing functions in wearable devices despite lacking sensors [13].

The multifunctional integration phase (1990s–early 2000s) introduced dedicated health-monitoring wearables with early biosensors. An early exemplar, the Polar Sport Tester (Polar Electro Oy, Kempele, Finland) pioneered wireless heart rate monitoring and underscored the potential of physiological tracking in personal health technologies [15]. Smart rings emerged in the late 2000s, with devices like the Oura Ring (Oura Health Ltd., Oulu, Finland) miniaturizing biometric sensing through integrated photoplethysmography (PPG) and accelerometers to monitor sleep, activity, and cardiovascular metrics—representing a breakthrough in unobtrusive, sensor-driven wearable technology [16,25].

**Figure 3:**
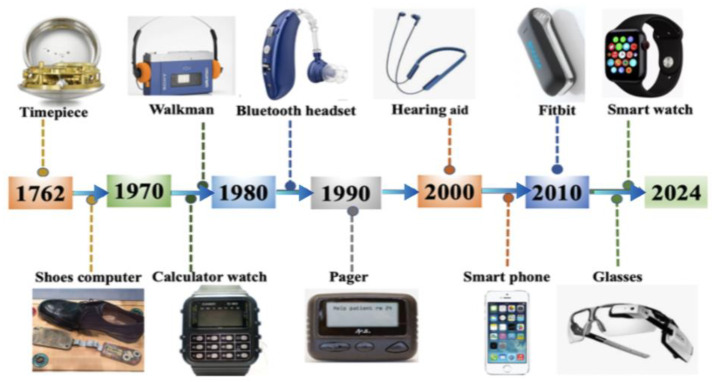
Evolution of wearable sensors over past five decades. Starting from 1760s, first wearable product was timepiece. Currently, wearable products are focusing on convenient use and information display.

The current smart technology phase (2010–present) features AI-enhanced wearable ecosystems, including EEG headsets and multimodal biometric monitoring systems [26,27]. Unlike earlier computational wearables such as calculator watches, modern devices function as comprehensive sensor systems that monitor real-time physiological and environmental parameters. These devices typically fall into two categories, those designed to enhance information acquisition through sensor optimization and data analysis, focusing on metrics such as sleep quality and activity tracking, and those aimed at increasing portability for multifunctional devices, emphasizing convenience and information display, as depicted in Figure 3.

### 2.3. Sensors and Sensing Data

In the interdisciplinary realm of human–machine interaction and behavioral studies, diverse sensor technologies are utilized to acquire detailed physiological and behavioral data. Eye-tracking devices, such as the EyeLink 1000 (SR Research Ltd., Mississauga, ON, Canada) and Tobii TX300 (Tobii AB, Danderyd, Sweden), precisely document ocular movements and focal points, offering critical metrics for analyzing reading patterns and visual attention tracking [28]. Similarly, gestural sensors like the Microsoft Kinect (Microsoft Corp., Redmond, WA, USA) and ASUS Xtion Pro (ASUSTeK Computer Inc., Taipei, Taiwan) are instrumental in capturing dynamic bodily motions [25], contributing to the development of interactive gaming environments and movement analysis. For spatial orientation, devices like FASTRAK (Polhemus Inc., Colchester, VT, USA) provide accurate locational data, indispensable for virtual reality applications.

Exploring the psychological and emotional dimensions, devices like the emWave System (HeartMath Inc., Boulder Creek, CA, USA) enable heart rate monitoring, while EEG instruments such as MindWave Mobile (NeuroSky Inc., San Jose, CA, USA), Neurosky Mindset (NeuroSky Inc., San Jose, CA, USA), and Enobio (Neuroelectrics, Barcelona, Spain) supply researchers with valuable insights into brain activity, advancing the understanding of cognitive processes and stress responses [26]. Additionally, the Empatica monitor (Empatica Inc., Boston, MA, USA) quantifies electrodermal activity (EDA), serving as a reliable indicator of emotional states and stress levels [29]. The integration of these wearable technologies has amplified their utility, particularly in the real-time and continuous monitoring of physiological states. Devices like Empatica often interface with electroencephalogram (EEG) and electrocardiogram (ECG) systems to record essential physiological signals, which, when paired with geospatial data from GPS systems, enable a contextualized analysis of health and behavioral patterns.

The convergence of these advanced instruments and their ability to collect multifaceted data enhances our understanding of individual behaviors and psychological states. This integrated approach not only deepens behavioral insights but also provides a comprehensive framework for the design of user-centric human–computer interaction systems, capable of addressing the complexities of real-world environments.

### 2.4. Multimode Sensor

In the field of educational technology, the integration of multimodal sensors represents a transformative advancement in understanding and enhancing learner engagement. For instance, Arroyo pioneered the analysis of multimodal data—such as facial expressions, head movements, gesture variations, and physiological responses—in computerized classrooms [12]. Similarly, Aguilera integrated system log files with facial recognition models to derive adaptive educational insights [24]. Their work extended to modeling interactive data from educational games using real-time annotation protocols, highlighting the reliability of these models in dynamic classroom contexts [15,16].

The development of deep learning technologies has further advanced learning analytics, significantly improving the precision of multimodal data modeling. For instance, Yun et al. utilized webcams and Kinect sensors to analyze skeletal movements and facial features, showcasing the efficacy of deep learning algorithms in engagement modeling [25]. While the exploration of biometric data, including ECG, EDA, and EEG, in engagement modeling remains in its early stages, studies suggest that biometric data offer superior precision in reflecting cognitive states and emotional arousal [26]. For example, Cohn et al. integrated facial recognition with biometric data, and Bandara et al. investigated cardiac data’s correlation with emotional engagement, with both affirming the enhanced accuracy and predictive reliability of multimodal fusion models and the need for comprehensive biometric datasets [27].

The synergistic effect of multimodal sensors in learning analysis lies in their ability to provide a holistic view of learner engagement by combining diverse data streams. For instance, the fusion of physiological data (e.g., ECG, EEG) with behavioral data (e.g., facial expressions, gestures) enables a more nuanced understanding of cognitive and emotional states. This integration allows for the identification of patterns that single-mode sensors might miss, such as the interplay between stress levels (captured by EDA) and attention (captured by EEG) [25,29].

Moreover, the fusion methods use for different sensor data play a critical role in enhancing learning outcomes. Techniques such as feature-level fusion, where raw data from multiple sensors are combined before analysis, and decision-level fusion, where outputs from individual sensors are integrated post-analysis, have shown promise in improving the accuracy of engagement models [27]. For example, combining facial expression data with physiological signals can help to distinguish between genuine engagement and superficial attention, thereby providing more reliable insights for educators [16,24].

The influence of multimodal sensor fusion on learning effects is profound. By leveraging complementary data sources, educators can design more personalized and adaptive learning experiences. For instance, real-time feedback from multimodal sensors can inform interventions, such as adjusting the difficulty of tasks based on a learner’s stress levels or providing targeted support when disengagement is detected [12,15]. This approach not only enhances learning efficiency but also fosters a more inclusive and responsive educational environment.

The integration of multimodal sensors into educational contexts has evolved from traditional regression models to deep learning approaches and emerging biometric applications, reflecting a progressive enhancement in learner engagement analytics. This evolution underscores the importance of continued research into multimodal fusion techniques and their practical applications, as they hold the potential to revolutionize how we understand and support learning processes [16,26].

## 3. Wearable Sensors for Learning Analytics

### 3.1. Overview of Prior Research on Wearable Sensors in Learning Analytics

To provide a structured overview of prior research on wearable sensors in learning analytics, representative studies were selected and analyzed in terms of their focus, methodological strengths, and limitations. The summarized literature reflects the current scope and diversity of research efforts in this field, serving as a foundation for identifying trends, gaps, and opportunities for further investigation. The key characteristics of these studies are presented in Table 2.

### 3.2. Learning Analytics

**Figure 4:**
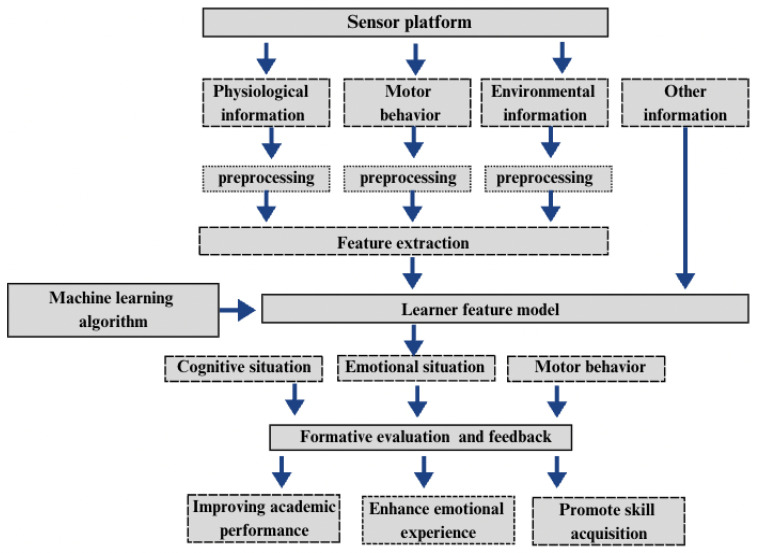
Learning analytics framework based on sensing data.

Learning analytics systematically measures, collects, analyzes, and reports learner-related data to enhance understanding and optimize learning processes [6,24]. As shown in Figure 4, wearable sensors play a crucial role by continuously capturing multimodal data, including physiological signals (e.g., EEG, ECG, EDA), motor behavior (e.g., accelerometers, gyroscopes), and environmental factors (e.g., temperature, noise levels) [35,36]. These data streams undergo preprocessing to remove noise and extract key features before being analyzed by machine learning models, which detect cognitive workload, engagement levels, and emotional states [5,25].

The extracted features contribute to a learner feature model that characterizes cognitive, emotional, and motor behavior states [26,27]. For example, EEG and ECG data help assess attention levels, while motion sensors detect engagement through physical activity. This model informs formative evaluation and adaptive feedback mechanisms, allowing for real-time interventions such as content adjustments, cognitive load regulation, and interaction-based engagement strategies [28]. If excessive stress is detected, the system may recommend a break, while reduced engagement may trigger interactive learning prompts.

This framework demonstrates how wearable sensors and learning analytics create a dynamic, data-driven feedback loop, enabling real-time pedagogical adjustments to improve academic performance, emotional well-being, and skill acquisition [2,12].

### 3.3. Sensor Data in Learning Analytics

The foundation of learning analytics lies in collecting, analyzing, and responding to data on learning behaviors and states. Sensors in smart educational environments and wearables enable extensive data collection, expanding analysis capabilities. While traditional methods focus on structured online behavior data, sensor technologies offer deeper insights into learners’ physical activities and emotional contexts. For instance, motion sensors discern learning states through behavioral analysis, while biometric sensors measure physiological indicators like heart rate and EEG signals to reveal emotional states and attention shifts [37,38]. Environmental sensors capture contextual data such as light and noise, shedding light on how external conditions influence concentration levels. Physiological indicators like heart rate and body temperature correlate with learning stress, cognitive load, and self-regulation capabilities [39,40]. Gesture and posture analysis support assessments of kinesthetic learning. Facial recognition techniques identify emotional states, aiding in recognizing engagement and frustration levels [17,41,42]. A galvanic skin response (GSR), reflecting changes in skin conductance due to stimuli, serves as a reliable physiological indicator of emotional arousal, aligning well with other emotional recognition methods [32]. The integration of diverse sensor data types has enhanced the understanding of learners’ knowledge acquisition and emotional attitudes.

In summary, sensor-based learning analytics collects multimodal data, constructs predictive models, and facilitates personalized guidance, offering a transformative approach to understanding and enhancing the learning process. Studies have employed wearable wristbands to monitor students’ physiological signals, such as heart rate and EDA, during classroom learning [43,44]. These data help educators identify engagement levels and emotional states, enabling timely instructional adjustments.

### 3.4. The Advantage of Wearable Sensors for Learning Analytics

**Figure 5:**
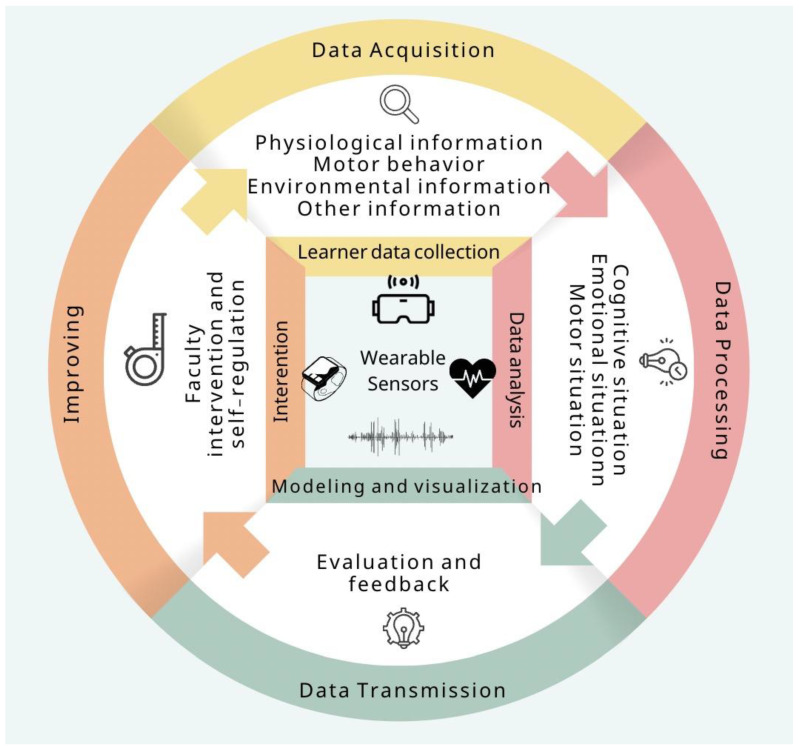
The use of learning analytics in wearable sensors in educational settings.

The widespread adoption of wearable sensors in formal educational settings is due to their well-established operational mechanisms, as illustrated in Figure 5. Research in these contexts often utilizes sensor data to evaluate learners’ experiences or outcomes [33,45] and to investigate how various instructional designs affect the effectiveness of instruction [46,47]. Beyond formal education, sensor-based analytics are utilized in informal learning scenarios, focusing on engineering projects, collaborative tasks, and venue-based experiential learning contexts [48,49]. Sensors play a crucial role in understanding internal psychological processes that are difficult to capture through traditional methods. They offer non-intrusive methods to continuously measure learners’ facial expressions, movements, and physiological signals. In certain studies, these signals have also been used to assess children’s cognitive functions, such as color recognition abilities [50]. The incorporation of sensor data, online learning metrics, and pedagogical information has further addressed challenges such as learner modeling, collaborative learning, and pedagogical analytics. Among these, biosensors, such as EEG and ECG, have been particularly effective in identifying cognitive workload and emotional engagement in students. EEG signals help detect attentional focus, while ECG patterns correlate with cognitive load and stress levels. Additionally, EDA fluctuations indicate emotional arousal, assisting in the real-time personalization of learning experiences. For example, when EEG readings suggest cognitive overload, an adaptive system may reduce task complexity to optimize learning retention and mental processing.

In the age of intelligence, digital learning environments have become crucial venues for education. Researchers leverage sensor data to study the impact of situational factors (e.g., multimedia material design, online learning platforms, and game-based learning) on educational outcomes [51,52,53].

## 4. Trends

### 4.1. The Need for Educational Development

Based on the literature, current trends in applying wearable sensors within learning analytics emphasize the real-time monitoring of students’ physical and cognitive states to evaluate and enhance their engagement and emotional well-being. Machine learning is a key tool in analyzing sensor data, identifying patterns in learning behaviors, and optimizing teaching methodologies [25]. Adaptive learning systems, which adjust content and difficulty based on real-time student feedback, are increasingly prevalent, fostering personalized and learner-centered experiences. Additionally, multimodal data fusion is used to increase the accuracy and scope of analyses, demonstrating the potential of wearable sensors in advancing personalized learning and promoting holistic student development. However, these technological advancements must align with the goals of educational development and respect the principles of human development, avoiding the indiscriminate application of technology in educational contexts. We identify several trends in education that should guide the integration of wearable sensors into learning analytics [27].

The “Transforming our World: The 2030 Agenda for Sustainable Development” outlines a vision for inclusive, equitable, and high-quality education that emphasizes lifelong learning and the elimination of gender disparities [54]. Within this framework, wearable devices have significant importance by enabling personalized learning experiences, facilitating access to education across diverse environments, and fostering immersive methodologies. These capabilities align with the agenda’s call for innovative technologies to enhance engagement and accessibility, thereby supporting inclusivity and equity in education worldwide. A report entitled “Artificial Intelligence and the Future of Teaching and Learning” [55] highlights a shift toward adaptive and personalized educational environments, emphasizing the ethical and equitable deployment of AI technologies. The integration of wearable devices with AI capabilities enhances their ability to provide personalized feedback and immersive learning experiences. However, frameworks like humans-in-the-loop stress the importance of ensuring that AI and wearable technologies complement human judgment rather than replace it. The concept of the “Long Tail of Learner Variability” further underscores the necessity for AI systems to address diverse learner needs, promoting inclusivity and equity. Wearable technology, while transformative, requires robust data privacy measures and equitable access policies to maximize its benefits responsibly.

UNESCO’s “Reimagining Our Futures Together: A New Social Contract for Education” advocates for an education system rooted in human rights, inclusivity, and lifelong learning, leveraging digital technologies to foster collaboration and solidarity [56]. This vision includes interdisciplinary and ecological curricula and emphasizes teaching as collaborative knowledge work. Wearable devices align with these objectives by enabling immersive, adaptive learning environments that accommodate diverse learner needs. However, their integration must adhere to ethical standards, safeguard privacy, and ensure equitable access, consistent with the report’s vision of education as a communal and publicly funded endeavor.

The OECD’s “Student Agency for 2030” framework highlights the importance of student agency in shaping educational experiences and fostering lifelong learning skills [57]. Central to this vision is co-agency, which emphasizes collaboration among students, educators, and communities to achieve shared educational goals. Wearable technologies can play a pivotal role in this framework, bridging formal and informal learning environments and promoting connectivity and inclusivity. Yet, their implementation must carefully consider ethical concerns, equitable distribution, and privacy protections to uphold education’s role as a public good and support holistic student development. Furthermore, frameworks such as the Framework for 21st Century Learning, Singapore’s 21st Century Competencies, and CASEL’s SESS competencies emphasize holistic education that integrates academic, social, emotional, ethical, and cognitive dimensions. This vision aligns with wearable devices’ ability to collect real-time physiological and behavioral data, such as heart rate variability or motion patterns, enabling personalized learning analytics. For instance, wearable sensors can monitor emotional states and engagement levels during collaborative tasks, providing immediate feedback to students and educators on areas requiring improvement. Unlike laptops or smart TVs, wearable devices are designed to operate unobtrusively, continuously capturing biometric and contextual data that facilitate adaptive learning interventions. This capability allows wearables to bridge formal and informal learning settings, offering real-time guidance tailored to individual learners’ needs. To ensure their potential is fully realized, the implementation of wearable technologies must adhere to ethical standards, safeguard data privacy, and promote equitable access.

### 4.2. Research of Wearable Sensors for Learning Analytics

The digital interaction logs of students using online educational platforms or Virtual Learning Environments (VLEs) have traditionally served as primary data sources for Transactional Analysis (TA) and learning analytics (LA). Recent advancements in wearable and mobile technologies have significantly enhanced the scope of educational data collection, enabling the continuous and non-intrusive measurement of physiological, cognitive, and behavioral states [4]. For instance, biosensors embedded in wearable devices collect heart rate variability, galvanic skin response, and motion data, offering insights into students’ stress levels, cognitive engagement, and emotional responses during learning activities. This advancement has also uncovered previously inaccessible data, such as real-time emotional fluctuations and micro-engagement patterns during collaborative tasks, which are difficult to measure using traditional methods like surveys or self-reports. Such granular data empower educators and researchers to design more adaptive and personalized learning interventions [34]. This evolution has driven a surge in research output, as well as the development of novel methodologies, models, and algorithms [30,31]. These innovations facilitate continuous, non-intrusive data collection, allowing researchers to assess complex educational metrics that were previously difficult to measure in real time [58].

In educational contexts, wearable technologies offer multifaceted benefits, as highlighted by Bower [14], including on-demand information access, improved recording, and feedback mechanisms [43], streamlined resource sharing [19], hands-free operation [23], first-person perspectives, interactive simulations, and enhanced engagement and immersion [59]. Research has increasingly focused on diverse technological tools such as motion detectors, eye-tracking devices, localization technologies, emotion recognition through facial analysis, and environmental monitoring tools that measure variables like temperature, atmospheric pressure, and carbon dioxide levels [60]. The integration of sensors with clothing, combined with the use of technologies like eye-tracking, EEGs, accelerometers, and audio–video recording systems, has further broadened the possibilities for data collection in classroom analytics. Analyzing this complex multimodal data requires interdisciplinary expertise spanning pedagogy, psychology, statistics, and signal processing [61].

Innovative visual tools and dashboards have been developed to translate multimodal data streams into actionable educational insights. For instance, prior studies on visual notation systems have demonstrated how various graphical elements—such as skill meters, emoticons, traffic lights, topic boxes, histograms, word clouds, and matrices—can be utilized to represent complex learning dynamics [62]. Additionally, affective and contextual analysis dashboards, such as the EMODA platform, have been employed to monitor student emotions and deliver real-time feedback aimed at enhancing teacher–student interactions [63]. These approaches are further supported by recent advancements in online and video-based learning platforms, which incorporate interactive video tools and open-source software to evaluate key learning variables [18].

Wearable sensors are increasingly recognized as transformative tools in education. They enable active and collaborative learning environments [64], foster physical engagement, enhance motivation [65], and support interactive pedagogical strategies for both students and teachers [66]. Giannakos has demonstrated that wearable technologies effectively monitor student engagement, providing detailed, dynamic insights into learning processes that refine educational systems and inform instructional design [28]. His research further highlights the role of wearables in supporting quantified self-technologies, promoting self-assessment and metacognitive reflection among learners [67]. Additional studies underscore the capacity of wearable sensors to integrate diverse data streams, enriching learning systems with features like adaptability and emotional recognition while enhancing learner engagement through embodied interaction and cognitive enhancement.

Havard’s research emphasizes the ongoing evolution of wearable technology in educational research, noting the increasing accessibility and sophistication of these devices [68]. This shift aligns with broader societal trends toward digital mobility, amplifying the potential of wearables to enhance learning in both structured and informal educational contexts. Wearable sensors provide a unique opportunity to refine research methodologies, offering unparalleled insights into learning behaviors, emotional states, and cognitive processes while fostering the development of adaptive, learner-centered educational systems.

### 4.3. Application of Wearable Sensors for Learning Analytics

Centered on the evolving demands of learners for enhanced learning experiences and effective instructional management within physical learning spaces, researchers have undertaken extensive empirical studies and system development in five key areas, as summarized in Table 3. These are intelligent tutoring systems, virtual learning companions, emotional interaction support, self-regulation ability assessment, and academic situation analysis and monitoring.

## 5. Challenges

### 5.1. Ethical Issues

In the domain of wearable sensors, research has frequently neglected the ethical considerations associated with their deployment, leaving critical concerns inadequately addressed [66,67,68,69]. Among the limited number of studies exploring ethical issues, three primary concerns have been identified: privacy, equality, and bias risks [70]. Privacy concerns predominantly relate to the unintended surveillance and potential mishandling of sensitive data collected by wearable devices, especially when these devices inadvertently capture information about individuals not directly involved in the study [71,72]. For example, wearable technologies may record personal data without obtaining proper consent, raising significant ethical questions about data ownership and user autonomy [20]. Beyond privacy, algorithmic bias presents another critical issue in learning analytics, particularly in the classification of students based on physiological responses. Machine learning models trained on limited demographic datasets may lead to misinterpretations of student engagement and cognitive states, potentially reinforcing biases in educational assessment. Additionally, discrepancies in sensor calibration and data standardization create inconsistencies that hinder interoperability across different educational platforms, raising concerns about data reliability and fairness.

Equality issues emphasize the challenge of providing equitable access to wearable sensor technologies for all educational stakeholders. The financial and logistical burden of adopting these devices can disproportionately affect resource-constrained environments, hindering their widespread implementation and potentially exacerbating existing educational inequalities [21,73]. Additionally, cultural imbalances in the research on wearable sensors pose another barrier, as most studies are conducted in English-speaking contexts, restricting the applicability of findings to non-English-speaking populations and further marginalizing diverse educational settings.

Bias risks arise from the predictive analytics employed in wearable sensor technologies, where demographic features such as gender or cultural background may inadvertently influence outcomes [69]. This is particularly problematic when unsupervised machine learning techniques classify students in ways that could reinforce stereotypes, harm self-esteem, or create biased expectations among educators [74,75]. Such risks underline the importance of ensuring fairness and transparency in the design and application of predictive models, as these classifications can have long-term implications for students’ educational experiences and outcomes.

To address these ethical concerns, specific solutions and frameworks must be implemented. For privacy protection, robust data anonymization techniques, such as differential privacy and k-anonymity, can be employed to ensure that individual identities are not discernible from the collected data [20,71]. Additionally, ethical review mechanisms, such as institutional review boards (IRBs) with expertise in wearable technology, should be established to evaluate the ethical implications of data collection and usage. These boards can enforce strict guidelines for obtaining informed consent, ensuring that participants are fully aware of how their data will be used and stored.

To tackle equality issues, funding initiatives and partnerships with technology providers can help subsidize the cost of wearable devices for under-resourced schools and communities. Furthermore, research should be expanded to include non-English-speaking populations, with collaborations between global institutions to ensure that findings are culturally inclusive and applicable across diverse educational contexts [21,73].

For bias mitigation, fairness-aware machine learning algorithms should be integrated into predictive models to detect and correct for demographic biases. Techniques such as adversarial debiasing and fairness constraints can help to ensure that classifications are equitable and do not perpetuate stereotypes [74,75]. The transparent reporting of model performance across different demographic groups should also be mandated to hold developers accountable for biases in their systems.

Addressing these ethical concerns is essential for the responsible integration of wearable sensor technologies in education. By implementing robust frameworks to safeguard privacy, promote equitable access, and mitigate bias, the benefits of wearable technologies can be realized without compromising fairness or causing unintended harm to learners. These measures will not only enhance the ethical integrity of wearable sensor research but also foster trust and acceptance among educators, students, and policymakers.

### 5.2. Explainable Learning Analytics

The integration of sensor data from physical learning spaces with log data from virtual environments marks a significant trend in learning analytics. In particular, current developments emphasize the integration of multimodal data, the diversification of spatial data sources, and enhanced precision in perceptual modeling. However, this convergence presents considerable challenges in designing learner models and managing data interaction. Successful applications that utilize sensor data to provide adaptive feedback during instructional processes remain limited, and data collected from wearable sensors often lack authenticity. Pope’s research highlights that wearable technologies can influence student behavior in areas such as health, sedentary habits, exercise routines, classroom participation, and lifestyle changes, introducing variability that complicates the interpretation of sensor data [76]. Furthermore, technical solutions for parallel perception and the integrated analysis of multimodal data, as well as unified learning systems enabling human–computer interaction and student self-reflection, are still underdeveloped.

The explanatory power of sensor data in terms of understanding learning patterns is constrained. While sensor data enrich learning analytics by capturing diverse physiological, behavioral, and environmental metrics, their capacity to elucidate learning phenomena remains limited. Predictive studies using multimodal data—such as eye movement, brain activity, heart rate, and body temperature—have shown low accuracy rates, with engagement predictions ranging from 12.08% to 39.44% and academic performance predictions between 6.21% and 25.49% [77]. Additionally, quantified self-data are prone to inaccuracies, whether due to user-logged errors or device inconsistencies. For instance, shaking a smartphone can register false steps, and metrics from different device brands often vary. These inaccuracies undermine the reliability of sensor data, rendering them more appropriate as auxiliary indicators rather than definitive metrics for educational or clinical decision-making. Empirical evidence linking sensor data—such as heart rate, blood pressure, and brain activity—to specific learning outcomes is also sparse, further impacting the effectiveness of sensor data in learning contexts.

Despite these limitations, research leveraging sensor data to optimize learning environments has achieved promising results. For instance, learning environment awareness systems designed with environmental sensors can create personalized learning pathways tailored to students’ real-world scenarios. Gesture recognition and interactive reading systems have also been shown to improve children’s interest in reading, enhancing their language and comprehension skills [71,72]. However, these interventions are relatively underdeveloped, with simplistic models and insufficient personalized feedback.

The integration of technical and educational metrics presents an additional challenge. Technical metrics prioritize precision and accuracy to meet technological requirements, while educational metrics emphasize learning efficiency and outcomes. This dichotomy complicates the adoption of wearable technologies for educational purposes. For example, while activity recognition algorithms are effective, they often lose accuracy in static recognition scenarios. Moreover, data quality degradation caused by sensor misalignment, omissions, and operational challenges further impacts the reliability of sensor data for learning analytics [20].

In terms of multimodal data, current methodologies are limited to basic fusion, aggregation, and statistical analysis, thereby underutilizing the potential of this rich domain [21]. Researchers have conducted numerous empirical studies on collecting multimodal data using wearable devices. For example, Su proposed a conceptual model categorizing multimodal data into digital, physical, physiological, psychometric, and environmental types, and linking them to learning indicators such as behavior, cognition, emotion, collaboration, and engagement [73]. Su also identified three integration methods: the one-to-many method (to improve measurement accuracy), the many-to-many method (to enhance informational richness), and the multimodal data validation method (to provide empirical evidence for integration). While these models offer valuable directions for future research, challenges remain in acquiring effective multimodal data, leveraging these data and theoretical models to infer learning states like emotions, cognition, and attention, and identifying the learning services that multimodal data integration can offer to students.

### 5.3. Technological and Data Challenges

Learning analytics focuses on the data-driven description, diagnosis, prediction, and intervention of learning processes. Despite its potential, several critical issues and challenges persist, particularly in analytics derived from sensor data. In data collection, methods often combine sensor data with self-reports to provide a comprehensive description of learning behaviors. However, these approaches struggle to balance unobtrusiveness with comprehensive accuracy, as sensor data alone cannot fully capture the nuanced context of learner behaviors and states. In model development, machine learning models frequently underperform due to suboptimal fit, with most relying on supervised learning techniques that require substantial labeled datasets, leading to significant costs and scalability issues. Feedback mechanisms, another vital component of learning analytics, are often simplistic and lack the personalization needed to address individual learner needs effectively. Pope’s research highlights how wearable technologies can influence behaviors beyond learning, such as health, sedentary lifestyles, exercise routines, classroom participation, and overall lifestyle habits, suggesting the need for models that can contextualize such behavioral changes [74].

The diversity of quantified self-tools and data types poses compatibility challenges, limiting the effective utilization of sensor data. Without proper integration and analysis, accumulated data often remain as fragmented figures with limited referential value. To derive meaningful insights, there is a pressing need for robust multi-source data integration and advanced analytical techniques to uncover underlying patterns in learners’ individual characteristics and their dynamic changes over time.

A significant limitation in current research is the lack of robustness to noise, particularly in classroom environments where factors like facial obstructions, lighting variations, and background distractions frequently interfere with data collection and modeling. Many existing systems function only under ideal conditions, underscoring the necessity of defining modeling constraints up front rather than relying solely on post hoc data cleaning to enhance precision. Additionally, the generalizability of models remains a challenge, as few studies consider their application across varying temporal and geographical contexts. Wearable sensors, being inherently mobile, exacerbate these issues. In dynamic environments, such as outdoor learning or physically active scenarios, physiological changes like heart rate spikes due to exercise can confound interpretations, as they may not directly correlate with stress or frustration in learning. This complexity highlights the critical need for context-aware algorithms capable of distinguishing learning-induced signals from other influences.

From a technical perspective, the computational complexity of processing multimodal sensor data and the associated storage requirements present significant challenges. For instance, real-time analysis of high-frequency physiological data demands substantial computational resources, while the long-term storage of such data can quickly become prohibitive in terms of capacity and cost. Efficient data compression techniques and edge computing solutions, where data are preprocessed locally on wearable devices, can help mitigate these challenges [74,75].

During the learning process, learners frequently experience negative emotions such as confusion, anxiety, boredom, or disappointment, which may arise from challenging content or disengaging instructional methods. Effective emotional perception is crucial to understanding learners’ needs and devising targeted instructional strategies. Without timely intervention, negative emotions can adversely affect learners’ cognitive states and knowledge acquisition. Personalizing such interventions—whether through human–computer interactions, teacher dialogue, or automated systems—requires continuous empirical research to determine when, where, and how these interventions should occur to maximize learner receptivity and outcomes.

Overemphasis on data control in educational contexts can also have unintended consequences. While the goal of collecting educational data, such as test scores and learning metrics, is to improve self-management and efficiency, discrepancies between expected and actual results can induce stress and anxiety among students and educators. For example, a student closely monitoring performance metrics may feel demotivated if their efforts do not translate into visible progress, potentially causing them to doubt their learning strategies or abilities. Conversely, over-reliance on quantitative educational metrics may lead some learners to prioritize data over professional guidance, fostering a mechanistic approach to education and neglecting the holistic and nuanced nature of cognitive development. Such overdependence risks obscuring learners’ true psychological needs, resulting in a loss of self-awareness and critical thinking, ultimately undermining the broader goals of education and personal growth [75]. A concise summary of the key challenges discussed in Section 5.1 to Section 5.3 is provided in Table 4, offering a structured perspective on current limitations in sensor-based learning analytics.

## 6. Future Directions

The forthcoming wave of technological revolution, fueled by big data, artificial intelligence, and smart sensing, is poised to bring transformative changes to educational environments, pedagogical approaches, content delivery, and assessment mechanisms. Big data, in particular, is increasingly permeating all facets of education, including teaching, learning, management, and evaluation, uncovering significant potential for optimizing and reimagining the educational ecosystem. This integration is driving profound shifts in the evolution of educational practices and research paradigms, bridging traditional methodologies with innovative solutions. Traditional classrooms, multimedia-enabled learning spaces, libraries, reading rooms, and outdoor educational environments are at a critical juncture, where digital transformation and intelligent systems are reshaping the boundaries of how and where learning takes place.

### 6.1. Safeguarding Data Privacy and Security

From a data ethics perspective, the intended use of data and the ethical boundaries of employing data-driven intelligent monitoring technologies must be carefully examined. Balancing the benefits and drawbacks of these technologies requires consideration of the perspectives of all stakeholders, including learners, parents, teachers, school administrators, and technology providers. Unlike academic performance metrics, students’ physiological data constitute sensitive personal information. These raw data typically do not hold direct value for instructional practices; instead, it is the actionable learning state information derived from these data that educators seek. Consequently, it is vital to identify the real beneficiaries of these technologies and define their access rights at various stages of data processing. Establishing robust privacy protection protocols entails specifying the types of data that should be collected and shared within the educational domain, assigning clear responsibility for safeguarding private data, and fostering secure and effective online practice communities. From a technological design perspective, protecting the rights of data producers is critical to preventing privacy intrusions without user consent. The LISA project exemplifies best practices by storing learners’ physiological perception data in personal web spaces, accessible only to devices capable of identifying the individual. This approach ensures that sensitive data are not disseminated across public networks or stored on anonymous cloud services. Instead, users retain full control over which data are shared for analysis by learning service systems and what information can be disclosed to teachers, parents, or peers. Furthermore, users are informed about the specific data collected on them, enhancing transparency and fostering trust. This ethical framework not only protects individual privacy but also empowers users to manage their data within a secure and informed context.

This direction is achievable in the short term, as it builds on existing data privacy frameworks and technologies. Solutions such as differential privacy, encryption, and secure data storage protocols are already available and can be adapted for educational contexts. The establishment of ethical guidelines and IRBs can also be carried out relatively quickly.

### 6.2. Establishing Comprehensive Biological Databases

Incorporating “posture” into multimodal engagement modeling represents a significant and underexplored research frontier. Existing studies have primarily focused on analyzing learners’ head positions, often relying on video recordings to capture motion, with the resolution being dependent on the type of feature extraction applied to the footage. Research on broader bodily movements remains limited and generally falls into two categories: work examining how various teacher bodily gestures, such as instructional gestures (e.g., tapping or pausing), affective gestures (e.g., fist clenching or arm swinging), physical proximity, and slight leaning during listening, influence learners, and work exploring the hierarchical significance of learners’ postures at different stages of the learning process, particularly in relation to their learning styles. From an embodied cognition perspective, posture, gestures, and motion are interconnected movement patterns often linked to acquired skills. Gestures, as the coordinated movements of multiple body parts, are typically intentional and function to provide immediate feedback loops (e.g., fist clenching as a sign of concentration) or to emphasize particular signals (e.g., head drooping in response to difficulty) during the learning process. Non-action skill movements, often unconscious, can reveal internal learner states, such as tension or skepticism, through physical instability or repetitive motions.

Physiological and bodily signals are reflective of rapid internal changes in learners, yet these reactions are often nonspecific and may result from social masking, making them difficult to interpret solely through external observation. Future research should focus on the interplay between learners’ biological attributes, psychological characteristics, and the learning environment, advancing the study of higher-order bodily and physiological signals within interactive contexts. Neuroscience and educational neuroscience techniques, such as brain–computer interfaces and infrared spectroscopy, could be pivotal in establishing causal links between student engagement and learning behaviors. This approach would integrate the synapse–neuron–neural network–biological system–learning behavior continuum to clarify the linkages between internal neurophysiological activity and external learning manifestations.

The development of a comprehensive biological database is foundational for creating robust deep learning models that predict learning engagement. Mapping physiological and modality data to engagement metrics is crucial, with eye gaze and central physiology providing insights into cognitive engagement, facial features and peripheral physiology representing affective engagement, and behavioral engagement emerging from interaction feature analysis. Currently, the availability of open, high-quality datasets for these purposes is limited. Even in the relatively advanced field of facial recognition, existing datasets are predominantly derived from non-educational scenarios and often exhibit cross-cultural or racial accuracy disparities [78]. These datasets also lack the granularity needed to capture learners’ transient micro-expressions and other nuanced behaviors during the learning process.

Establishing an open, shared, and collaboratively developed local database of learner biological data is an urgent and meaningful endeavor that may address these gaps. Such a database would enable researchers to overcome the limitations of existing datasets, facilitate cross-cultural inclusivity, and advance the field by supporting the development of models capable of accurately representing the complex interplay of physiological, affective, and behavioral signals in real-time learning scenarios. This initiative would not only enhance the precision of multimodal engagement modeling but also provide invaluable resources for interdisciplinary research across educational technology, neuroscience, and data science.

This is a medium-term goal. While the collection of physiological and behavioral data is feasible with current technologies, creating a comprehensive, open, and culturally inclusive database requires significant collaboration, funding, and standardization efforts. Challenges include ensuring data quality, addressing ethical concerns, and fostering international cooperation.

### 6.3. Advancing Multidisciplinary Collaborative Research to Construct Systemic Theoretical Models

The continued advancement of technology hinges on the cross-fertilization of multiple disciplines and the convergence of ideas from diverse fields. Future research in learning analytics should prioritize multidisciplinary collaboration to comprehensively mine and analyze sensor data, aiming to construct a systematic theoretical framework. This requires the integration of expertise from disciplines such as education, psychology, neuroscience, and neurobiology to leverage their theoretical foundations and methodologies. By drawing on insights from these fields, researchers can identify and extract learning-relevant indicators or dimensions from various sensor data types, such as cognitive, behavioral, emotional, and physiological metrics.

Establishing robust computational methods and weighting mechanisms for these indicators is essential to develop systematic analysis models capable of accurately interpreting complex learning phenomena. Such models would provide a stronger theoretical basis for empirical research, allowing for more precise and meaningful exploration of learning processes. This interdisciplinary approach not only enhances the analytical power of learning analytics but also ensures that research outcomes are both theoretically grounded and practically impactful, paving the way for innovations that bridge theoretical inquiry with real-world educational applications.

This direction is a medium- to long-term endeavor. While interdisciplinary collaboration is already underway in some areas, constructing a unified theoretical framework requires sustained efforts to integrate diverse methodologies and perspectives. This process will involve extensive research, validation, and iterative refinement.

### 6.4. Identifying Perception Data Suitable for Learning Analytics

The use of sensor-obtained perception data for educational data mining has emerged as a significant trend, driving experimental studies to determine which types of perception data are most suitable for learning analytics and capable of producing meaningful outcomes. To accelerate advancements in this field, the increased sharing of learning perception data by research institutions is essential, especially given the high costs associated with generating such data, which often involves deploying extensive sensor equipment, recruiting large cohorts of participants, and dedicating substantial time to experimentation. The use of perception-driven learning analytics in physical learning environments presents promising opportunities for adaptive modeling and the advancement of responsive learning systems. However, current technologies for measuring and analyzing learning states, including emotions, cognition, and learning behaviors, remain inadequate for accurately assessing classroom teaching quality.

Several challenges hinder the precision of such technologies. First, the inherent complexity of classroom environments, including variations in students’ postures and diverse teaching contexts, reduces measurement accuracy, rendering many current tools insufficient for the nuanced analytical demands of classroom instruction. Second, as educational reforms evolve, the scope of classroom teaching assessments is broadening. Beyond evaluating teachers’ effectiveness in transmitting knowledge, there is increasing emphasis on assessing their ability to foster collaborative knowledge construction among students. Addressing these multifaceted challenges requires both technological innovation and the redefinition of assessment criteria, aligning them with the dynamic and interactive nature of modern educational practices.

This is a short- to medium-term goal. Researchers can begin identifying relevant perception data types using existing sensor technologies and experimental setups. However, refining these datasets and ensuring their applicability across diverse learning contexts will require additional time and resources.

### 6.5. Enhancing the Accuracy of Emotional State Recognition

Sensor monitoring captures students’ physiological signals, offering sensitivity that surpasses traditional knowledge and skill assessments computed by learning agents. This heightened sensitivity necessitates careful consideration regarding the type of information teachers and parents should access from such personal data. While current sensor-based emotional detection technologies have yet to achieve optimal results, their potential for designing next-generation intelligent educational systems is significant. Traditional assessments may provide insight into the correctness of students’ answers but fail to uncover deeper dimensions of their emotional experiences during system use or classroom learning. Future wearable devices are expected to advance emotional detection capabilities, offering functionalities such as learning interventions and activity recommendations aimed at emotional regulation and interactive support. For instance, by detecting signs of frustration, these systems could predict when a learner requires rest or adjustments, allowing the learning environment to adapt dynamically to psychological states. This adaptation could foster learners’ motivation, enhance self-awareness in emotional regulation, and go beyond current health monitoring features. Learners exhibiting low mood or negativity in physical learning spaces could receive tailored guidance and emotional support from intelligent learning systems, leveraging wearable technology to maintain engagement and well-being [79].

However, future learning scenarios present challenges in accurately representing students’ emotional states due to potential influences from prior experiences or concurrent environmental factors. For example, physiological changes caused by physical activity during outdoor learning could confound emotional measurements, potentially introducing biases in the monitored data. To address this, research must explore methods for discerning signals specifically related to learning states. Using professional motion monitoring sensors to filter out physiological signals unrelated to learning could significantly enhance the precision and applicability of emotional state recognition in ubiquitous learning environments. Positive emotions have been shown to facilitate learning, particularly in solving creative problems, as noted by Isen and others [80]. Interestingly, emotions traditionally considered negative, such as confusion or tension, may also enhance learning under certain conditions, only becoming detrimental when they escalate beyond manageable levels. This underscores the importance of responsive systems capable of detecting persistent negative emotional states and providing timely, personalized feedback and regulation. Such advancements would ensure that learners receive appropriate support, maximizing the benefits of emotional data in fostering an adaptive and empathetic educational experience.

Notably, the latest machine learning and deep learning techniques, such as convolutional neural networks and transformer models, have shown promise in improving emotional state recognition by analyzing physiological and behavioral data. However, challenges like dataset scarcity, model generalizability, computational complexity, and interpretability limit their practical application. Techniques like edge computing and explainable AI are being explored to overcome these barriers, but further research is needed to ensure these solutions are effective and inclusive across diverse educational contexts.

This direction is achievable in the medium term. Advances in machine learning and sensor technologies are improving emotional recognition capabilities, but challenges such as noise reduction, context-awareness, dataset scarcity, and cultural variability must be addressed. Further research and development are needed to achieve optimal accuracy.

### 6.6. Immersive Learning Experiences and Non-Intrusive Sensing

Knowledge acquisition depends on a seamless learning process and a positive learning experience. To create an immersive learning environment, wearable technology must automatically sense contextual information, physiological signals, motion, and learning-related data surrounding the learner [5]. Integrating portable smart devices with wireless interfaces, cloud computing, and big data analytics facilitates precise and intelligent learning support [81]. However, challenges arise when learners, aware of being monitored, consciously or unconsciously alter their emotional states to appear more engaged. This behavior can lead to inaccuracies in emotion recognition and computational outcomes, thereby diminishing the effectiveness of learning analytics. Moreover, learners who highly value their privacy may resist the use of sensing devices, leading to disengagement and potentially hindering their participation in the learning process. Thus, while perception technologies hold promise, their introduction may negatively impact learning if these challenges are not addressed.

The integration of immersive learning experiences with non-invasive sensing technologies presents significant potential for transforming education [82]. Immersive learning environments simulate or create realistic scenarios that promote comprehensive engagement, encompassing deep sensory involvement—visual, auditory, and tactile—while fully activating learners’ emotions and cognition [83]. This holistic approach enhances deep understanding and supports long-term memory retention. Non-invasive sensing technologies further enrich these environments by continuously and unobtrusively capturing detailed physiological and behavioral data, allowing the dynamic monitoring of learning states and environmental factors without requiring active learner participation. When applied through wearable devices, these technologies enable educational researchers to identify subtle shifts in learning processes, such as cognitive load, emotional fluctuations, and attention allocation, with remarkable precision and granularity. By supporting the identification of key learning indicators, these innovations enable the design of more personalized, adaptive, and pedagogically effective interventions, ensuring that learners’ engagement and understanding are both maximized and authentically measured.

This is a long-term goal. While immersive technologies like virtual reality (VR) and augmented reality (AR) are advancing, integrating them with non-intrusive sensing for seamless learning experiences requires significant innovation. Challenges include minimizing learner discomfort, ensuring data accuracy, and developing adaptive systems that respond to real-time physiological and behavioral cues.

### 6.7. Leveraging Data from the Learning Process to Create Meaningful Educational Contexts

In contemporary school education systems, the exploration of instructional effectiveness in complex and open-ended scenarios necessitates the collection of extensive learning process data. These data are critical for adapting and conducting comparative analyses of teaching situations. Intelligent sensing technologies, particularly in classroom settings, are increasingly employed to observe student performance during activities such as group collaboration discussions and the use of smart tutoring and diagnostic systems. These technologies capture data on student attention, facial expressions, head posture, and gestures, which are processed through backend classroom data recording systems to identify learners who may require assistance. While such real-time classroom data capture technologies are already used in several Asian countries, these observations are machine-generated rather than teacher-driven. Effective adaptation and optimization of teaching still require teachers to interpret classroom dynamics and integrate learning perception data via manual analysis to design instructional scenarios that foster student motivation, positive emotions, and heightened engagement.

The technological revolution has introduced a specialized division of labor, creating the need for a large-scale, professionalized workforce to manage the reshaped learning environments. Intelligent sensing technologies, powered by the Internet of Things (IoT), smart perception, and machine learning, enhance data collection capabilities to unprecedented levels of depth and breadth. Combined with artificial intelligence, perceptual computing, and mobile connectivity, these technologies facilitate comprehensive quantitative analyses and multidisciplinary research into the drivers of educational processes. As class sizes grow, teachers face challenges in swiftly identifying and supporting individual learners within limited timeframes. Intelligent technology bridges this gap by providing tools for the precise analysis and prediction of students’ emotional and cognitive states. Automated suggestions and real-time feedback from computational systems can offer learning support to individuals experiencing emotional challenges, such as discouragement, boredom, or confusion. By offloading routine tasks to technology, teachers can refocus on the essence of educational service, dedicating their efforts to the emotional, physical, and moral development of their students.

However, the adoption of intelligent technologies must be carefully managed to prevent politicization. Schools should have the autonomy to select wearable technologies that align with their specific developmental needs, ensuring that technological integration supports their unique goals and contexts. Zhu identifies ten essential characteristics for intelligent learning environments, which serve as a framework for creating rich and seamless educational experiences: 1. location-aware systems that track learners’ real-time geographical positions; 2. context-aware capabilities to discern varied scenarios and activities; 3. social awareness to recognize and integrate social connections; 4. interoperability for compatibility across diverse resources, platforms, and services; 5. seamless connection ensuring uninterrupted interactions across devices; 6. adaptability to customize learning materials based on individual preferences and needs; 7. ubiquity in order to anticipate and clearly present learner demands, enabling transparent access to educational resources; 8. whole-record functionalities to document comprehensive learning trajectories for detailed analysis and personalized feedback; 9. natural interaction through multimodal engagement, such as facial expression and location recognition; 10. high engagement to create immersive and interactive learning experiences within technologically enriched environments [84].

These elements lay the foundation for intelligent learning environments that deliver enriched, individualized, and continuous educational experiences across both formal and informal learning settings. By leveraging wearable sensors and advanced learning analytics, future intelligent systems provide precise and comprehensive educational services, fostering a more personalized and seamless learning journey. This vision underscores the potential of integrating diverse technologies to transform education, aligning it with the needs of learners and the dynamic demands of modern educational landscapes.

This is a medium-term goal. While data-driven insights are already being used to personalize learning, creating meaningful educational contexts requires deeper integration of data analytics with pedagogical strategies. This will involve developing new tools and frameworks for educators to interpret and apply data effectively.

As summarized in Table 5, future research on wearable sensors for learning analytics will require innovation across technical, theoretical, and pedagogical dimensions.

## 7. Conclusions

Education must evolve in a more human-centered direction, transcending the passive role of technological surveillance using artificial intelligence, in order to foster the continuous refinement of the character, values, and identities of both educators and learners. This shift represents an essential choice for the deep integration of artificial intelligence into education, precise pedagogical practices, and the construction of a new educational ecosystem amidst the technological revolution. The use of learning analytics grounded in sensor data offers the potential to collect extensive educational data within physical learning environments while simultaneously supporting online and mobile learning in real-world contexts. This dual capability paves the way for innovative applications of learning analytics, bridging the divide between traditional and emerging educational paradigms.

This study highlights the current state, core content, and existing challenges in the application of sensor data to learning analytics. Based on these findings, it provides targeted recommendations and outlines future research directions, aiming to serve as a valuable reference for advancing both theoretical exploration and practical application in this field. Looking ahead, interdisciplinary collaborative research will be instrumental in developing robust theoretical frameworks, while empirical studies on personalized interventions will play a critical role in refining practical applications. By leveraging the combined strengths of artificial intelligence and human wisdom, educational researchers and practitioners can gain deeper insights into learning phenomena, uncover nuanced learning patterns, and optimize the learning process. This approach promises to create more adaptive, inclusive, and impactful educational experiences, aligning technological advancements with the humanistic goals of education.

## Figures and Tables

**Table 1:** The Classification and Application of Sensors.

Classification	Specific Type	Measured Data	Application Example
Motion sensor	Accelerometer	Accelerated velocity	Motion recognition and falling monitoring [13,14]
	Gyroscope	Angular velocity	
	Compass	Magnetic declination	
	Dynamometer	Force	
Biosensor	EEG	Brain wave	Expression recognition, pressure perception, attention change, and psychological health monitoring [15,16,17,18]
	Glucometer	Blood sugar content	
	ECG	Cardiac electrical activity	
	EDA	Electro dermal activity	
	Eye tracker	Blink and focus the pupils	
Environmental sensor	GPS	Location information	Environmental information, scenario inference, and smart home [19,20,21]
	Air quality sensor	Air quality	
	Thermometer	Temperature	
	Hygrometer	Humidity	
	Barometer	Air pressure	
Others	Optical camera	Optical imaging	Motion recognition and emotional judgment [22,23]
	Infrared camera	Thermal imaging	
	Microphone	Acoustical signal	
	Software sensor	Human-computer interaction data	

**Table 2:** Key characteristics of selected studies on wearable sensors in learning analytics.

Author(s) & Year	Focus	Strength	Weakness
Bandara et al., 2016, [27]	Explored the integration of physiological signals into affective learning analytics.	Demonstrated the complementary value of multimodal physiological data in emotion modeling.	Limited ecological validity and insufficient consideration of longitudinal effects.
Blikstein & Worsley, 2016, [30]	Proposed a framework for integrating multimodal sensor data into learning analytics.	Provided a theoretical foundation for computational sensing in complex learning tasks.	Lacked empirical implementation and omitted ethical and technical constraints.
Ochoa & Worsley, 2016, [31]	Discussed the role of sensor-based data in enhancing traditional learning analytics.	Emphasized the analytic potential of multimodal wearable data for real-time learning support.	Absence of validated models and underdeveloped pedagogical interpretation strategies.
Pijeira-Díaz et al., 2018, [32]	Applied biometric indicators to infer cognitive-affective states in learning contexts.	Demonstrated the feasibility of using biosignals for in-situ engagement detection.	Data reliability affected by environmental confounds and signal ambiguity.
Buchem et al., 2019, [5]	Conceptualized wearable-enhanced learning and its pedagogical affordances.	Mapped theoretical linkages between wearables, personalization, and feedback in education.	Lacked empirical evidence and specific sensor-level analysis.
Giannakos et al., 2020, [33]	Investigated the use of commercial wearables for tracking learner engagement.	Highlighted the practicality and institutional viability of wearable-based data collection.	Limited signal resolution and insufficient representation of cognitive processes.
Sharma & Giannakos, 2020, [34]	Examined the theoretical integration of multimodal data into educational analytics.	Advanced a multidimensional framework for interpreting learning signals.	No empirical validation and limited contextual applicability.
Khosravi et al., 2022, [3]	Studied biosensor-based methods to monitor learning dynamics in higher education.	Provided structured insights into sensor-informed adaptive feedback mechanisms.	Narrow participant scope and insufficient exploration of interoperability issues.
Ba & Hu, 2023, [12]	Reviewed emotional sensing via wearable devices in educational settings.	Synthesized sensor types, application scenarios, and signal modalities across studies.	Lacked methodological critique and discussion of deployment feasibility.
Shen et al., 2024, [11]	Empirically analyzed multimodal sensor integration for real-time learner modeling.	Validated a full sensor-based framework for continuous monitoring in education.	Underdeveloped treatment of ethical risks and explainability in learning analytics.

**Table 3:** An overview of the application of wearable sensors for learning analytics in thearticles reviewed.

Application	Utilized Technology	Function
Intelligent Tutoring Systems (ITS)	Intelligent Tutoring Systems	ITS determines students’ emotional states in real-time and provided adaptive support, resulting in enhanced learning outcomes through long-term usage.
Virtual Learning Companion (VLC)	Smart Monitor sensors and “Learning Companion” system	VLC provides personalized learning feedback to students and activated specific motivational mechanisms to encourage learners in completing difficult tasks.
Emotional Interaction Support (EIS)	Wireless networks and sensor technologies	EIS enhances students’ social interaction skills, promoted self-awareness and reflection on emotions, and assisted teachers in organizing classroom activities and adjusting teaching strategies.
Self-Regulation Skills Assessment (SSA)	Learning Management Systems	SSA offers an effective means to assess learners’ self-regulatory abilities
Academic Situation Analysis and Monitoring (ASAM)	Real-time monitoring	ASAM facilitates real-time emotional feedback and intervention for students potentially at risk of academic difficulties.

**Table 4:** Key challenges in applying wearable sensors for learning analytics.

Challenge Category	Description	Implications for Learning Analytics
Ethical Issues (5.1)	Includes privacy breaches, lack of informed consent, demographic bias, and unequal access to sensor technologies.	Undermines trust, excludes marginalized populations, and introduces systemic bias in learner classification and feedback models.
Interpretability and Validity (5.2)	Sensor-derived data often lack explainability and are affected by non-learning-related variables, limiting their educational interpretability.	Reduces the transparency of learning analytics outputs, impeding teacher adoption and potentially leading to misinformed interventions.
Limited Predictive Accuracy (5.2)	Physiological signals show weak and inconsistent correlations with learning outcomes, and sensor outputs are susceptible to error and device variation.	Challenges the reliability of engagement or performance predictions, making data suitable only as secondary indicators.
Data Integration and Technical Alignment (5.2–5.3)	Current systems face difficulties in combining multimodal sensor streams, aligning them with learning models, and maintaining coherence with digital platforms.	Hinders the implementation of real-time adaptive systems and comprehensive learner profiling.
Sensor Reliability and Environmental Robustness (5.3)	Signal distortion due to classroom noise, device misalignment, and learner movement reduces data quality in dynamic learning settings.	Compromises the generalizability and robustness of sensor-based learning analytics in authentic educational environments.
Computational and Implementation Barriers (5.3)	Real-time data processing requires significant computational power and storage; cost and system complexity hinder large-scale deployment.	Limits practical scalability, especially in under-resourced institutions, and increases reliance on edge-computing architectures.

**Table 5:** Future research directions for wearable sensors in learning analytics.

Future Direction	Core Focus	Development Outlook
Analyzing High-Order Physiological and Bodily Signals (6.1–6.2)	Investigate the links between learner posture, gestures, and internal states by integrating embodied cognition with neuroscience-informed approaches.	Medium-term: Requires interdisciplinary methods and interactive experimental paradigms.
Developing Inclusive Biological Databases (6.2)	Build open, granular, and culturally diverse databases of physiological and behavioral data for modeling cognitive and affective engagement.	Medium-term: Technically feasible but demands ethical oversight, international collaboration, and standardization.
Constructing Multidisciplinary Theoretical Frameworks (6.3)	Integrate theories from education, psychology, neuroscience, and computing to systematize multimodal sensor data interpretation.	Medium- to long-term: Calls for iterative model development and sustained cross-disciplinary research.
Identifying and Optimizing Perception Data (6.4)	Determine which types of sensor-derived perception data are most effective for assessing teaching quality and learner states.	Short- to medium-term: Experimental validation needed under authentic and complex classroom conditions.
Enhancing Emotional State Recognition (6.5)	Improve accuracy and reliability of emotion detection through advanced machine learning and signal filtering, with contextual awareness.	Medium-term: Requires large-scale datasets, explainable AI models, and bias reduction strategies.
Integrating Immersive Experiences with Non-Intrusive Sensing (6.6)	Combine virtual and augmented reality with continuous physiological sensing to create adaptive and authentic learning environments.	Long-term: Demands technological innovation, real-time response systems, and learner-centered design.
Creating Intelligent, Context-Rich Learning Environments (6.7)	Leverage data from the learning process to construct personalized and seamless instructional contexts guided by intelligent systems.	Medium-term: Requires new frameworks to align sensor analytics with pedagogical goals.
